# Mutations in Specific Structural Regions of Immunoglobulin Light Chains Are Associated with Free Light Chain Levels in Patients with AL Amyloidosis

**DOI:** 10.1371/journal.pone.0005169

**Published:** 2009-04-13

**Authors:** Tanya L. Poshusta, Laura A. Sikkink, Nelson Leung, Raynell J. Clark, Angela Dispenzieri, Marina Ramirez-Alvarado

**Affiliations:** 1 Department of Biochemistry and Molecular Biology, College of Medicine, Mayo Clinic, Rochester, Minnesota, United States of America; 2 Division of Nephrology and Hypertension, Mayo Clinic, Rochester, Minnesota, United States of America; 3 Laboratory Medicine and Pathology, Mayo Clinic, Rochester, Minnesota, United States of America; 4 Division of Hematology, Mayo Clinic, Rochester, Minnesota, United States of America; Griffith University, Australia

## Abstract

**Background:**

The amyloidoses are protein misfolding diseases characterized by the deposition of amyloid that leads to cell death and tissue degeneration. In immunoglobulin light chain amyloidosis (AL), each patient has a unique monoclonal immunoglobulin light chain (LC) that forms amyloid deposits. Somatic mutations in AL LCs make these proteins less thermodynamically stable than their non-amyloidogenic counterparts, leading to misfolding and ultimately the formation of amyloid fibrils. We hypothesize that location rather than number of non-conservative mutations determines the amyloidogenicity of light chains.

**Methodology/Principal Findings:**

We performed sequence alignments on the variable domain of 50 κ and 91 λ AL light chains and calculated the number of non-conservative mutations over total number of patients for each secondary structure element in order to identify regions that accumulate non-conservative mutations. Among patients with AL, the levels of circulating immunoglobulin free light chain varies greatly, but even patients with very low levels can have very advanced amyloid deposition.

**Conclusions:**

Our results show that in specific secondary structure elements, there are significant differences in the number of non-conservative mutations between normal and AL sequences. AL sequences from patients with different levels of secreted light chain have distinct differences in the location of non-conservative mutations, suggesting that for patients with very low levels of light chains and advanced amyloid deposition, the location of non-conservative mutations rather than the amount of free light chain in circulation may determine the amyloidogenic propensity of light chains.

## Introduction

Amyloidosis is a devastating group of disorders in which normally soluble proteins misfold and aggregate to form insoluble amyloid fibrils. Deposition of these amyloid fibrils leads to cell death and tissue degeneration. To date, more than 20 different proteins and polypeptides have been identified in disease associated amyloid deposits. These proteins include the Aβ peptide in Alzheimer's disease, immunoglobulin light chains in light chain or primary systemic amyloidosis (AL), and the islet-associated polypeptide in type II diabetes, among others [1], [2]. AL is the result of a clonal proliferation of monoclonal plasma cells in the bone marrow. These plasma cells synthesize high amounts of monoclonal immunoglobulin free light chains (LCs), also known as Bence Jones proteins (BJP). LCs are secreted into circulation and excreted in large amounts in urine. While in circulation, the LCs misfold into amyloid fibrils which in most cases (85%) are composed of the N-terminal variable domain [3]. The amyloid fibrils can be deposited in any visceral organ leading to organ failure and death.

A LC is composed of an N-terminal variable domain (VL) and a C-terminal constant domain (CL). The VLs are not uniformly variable throughout their lengths. Three small regions, the hypervariable regions or complementarity determining regions (CDR), show much more variability than the rest of the domain. These regions vary both in size and in sequence among different VL germline isotypes. These are the regions that determine the specificity of the antigen-antibody interactions. The remaining parts of the VL, four framework regions (FRs), have quite similar amino acid sequences.

**Figure 1:**
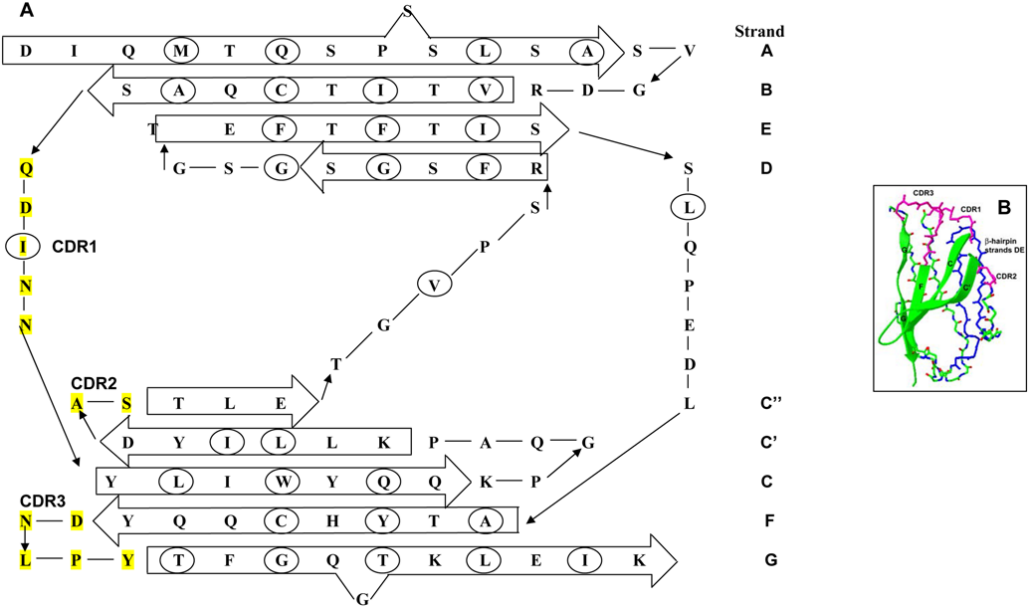
VL structure. A) Topological Diagram of the protein structure for AF490909 adapted from Schiffer, et al [28]. The two β-sheets of the domains have been separated. Residues that point towards the core are circled. CDR segments are highlighted in yellow. The β-strands have been encased within the arrows and are connected to their respective loops. B) Structural model of a VL (1BRE.pdb) showing CDR regions in pink, β-hairpin between strands D and E in blue and dimer interface in green ribbons.

The overall structure of the VL is an immunoglobulin fold with 9 β-strands (A, B, C, C′, C″, D, E, F, and G) packed tightly against each other in two antiparallel β sheets joined together by a disulfide bridge. The N- and C- termini strands (A and G, respectively) are parallel [4]. The topology is a form of a Greek key β-barrel. The CDRs form three loops between amino acids 24–34, 50–56 and 89–95 that contain the amino acids that will recognize the antigen (Figure 1). Immunoglobulin quaternary structure consists of two heterodimers formed by the LC and the immunoglobulin heavy chain (HC) interacting together via disulfide bonds. The LC VL domain interacts with the HC variable domain through β-strands C, C′, F and G. The source of sequence variability in LCs comes from combinatorial pairing of the V genes (40 κ and 33 λ) and the J genes (corresponding to strand G or FR4), making it possible to generate about 3000 different LC sequences. In addition, further sequence variation appears from somatic mutations to improve the affinity of the antibody for the antigen.

In AL, λ is overrepresented (3∶1) as compared to healthy individuals or multiple myeloma patients (λ/κ = 1∶2), especially the λ VI subtype [5]. In addition, VL germline donor gene usage in AL is biased [6], [7]. The three studies by Comenzo, Abraham and Prokaeva agree that in AL, the VL germline donor gene usage comprises Vλ II 2a2, Vλ III 3r, Vλ VI 6a, Vκ I O18/O8, while there are slight differences in the sample size, sample selection and the frequency of use of each germline donor gene in each study. Comenzo and co-workers demonstrated 30% of AL VL genes used Vλ VI 6a germline donor [6]. Abraham and co-workers found that most κ patients selected for their study used the Vκ I subgroup (77%) [7]. A similar observation has been made by Prokaeva and co-workers [8].

Current evidence suggests that AL proteins are less stable than their non-amyloidogenic counterparts [9], [10]. There are several possible sources of protein destabilization for AL proteins: 1) somatic mutations that cause the protein to sample partial unfolded states, 2) proteolytic cleavage that removes the constant domain, and 3) loss of the interaction with the HC due to mutations or truncations in LC or HC. Somatic mutations have a global destabilizing effect on AL proteins and as a consequence these proteins require less energy to unfold [11]–[13]. The propensity to form amyloid fibrils *in vitro* for some VLs appears to be inversely correlated with their free energy of unfolding, suggesting that both stabilizing and destabilizing interactions within the VL domain can influence the kinetics of amyloid formation [9], [10], [14]. The goal of our study was to determine the nature and the location of mutations in κ and λ VL sequences from AL patients and to identify patterns in the location of non-conservative mutations that correlate with clinical parameters, such as serum free light chain levels, that may help predict rate of amyloidogenesis.

## Results

A total of 46 AL sequences from Mayo, 48 AL sequences from Comenzo, and 47 AL sequences from Prokaeva were used for this study (for a detailed description of the sequences used, see the Methods section). Analysis of the mutational ‘hot spots’ was performed separately for AL κ and AL λ light chains. Figure S1 and Figure S2 show the sequence alignments of 50 AL Vκ protein sequences. As expected, the CDR regions of these VL proteins have accumulated a large number of somatic hypermutations. For the Vκ proteins in particular, positions 30 (CDR1), 93 (CDR3) and 53 (CDR2) as well as 70 (FR3, β-strand E), to a lesser extent, appeared to be mutational hotspots. No clear bias is observed in terms of amino acid substitution. For the 91 AL Vλ proteins, Vλ I–III sequences (Figure S3 and Figure S4) were analyzed separately from the Vλ VI (Figure S5) sequences. Somatic hypermutations accumulate in different positions depending on the germline. Vλ I positions 27 (CDR1), 38 (FR2, β-strand C), 50 (FR2), 52 (CDR2), and 95a (CDR3) appear to be mutational hotspots. Vλ II positions 47 (FR2, β-strand C′), 52 (CDR2), 89 (FR3, β-strand F) and positions 92–95b (CDR3) show a predominance in somatic hypermutation. Vλ III positions 31a (CDR1), 52 (CDR2), and 95a (CDR3) show great variability. Finally, the Vλ VI sequences show mutations in positions 43 (FR2), 52 (CDR2), and in the FR4 region between 95ab–96. All of the control sequences analyzed in this study showed less prevalence of mutational ‘hotspots’ in the positions found for AL sequences (Figure S6, S7, S8, S9).

**Figure 2:**
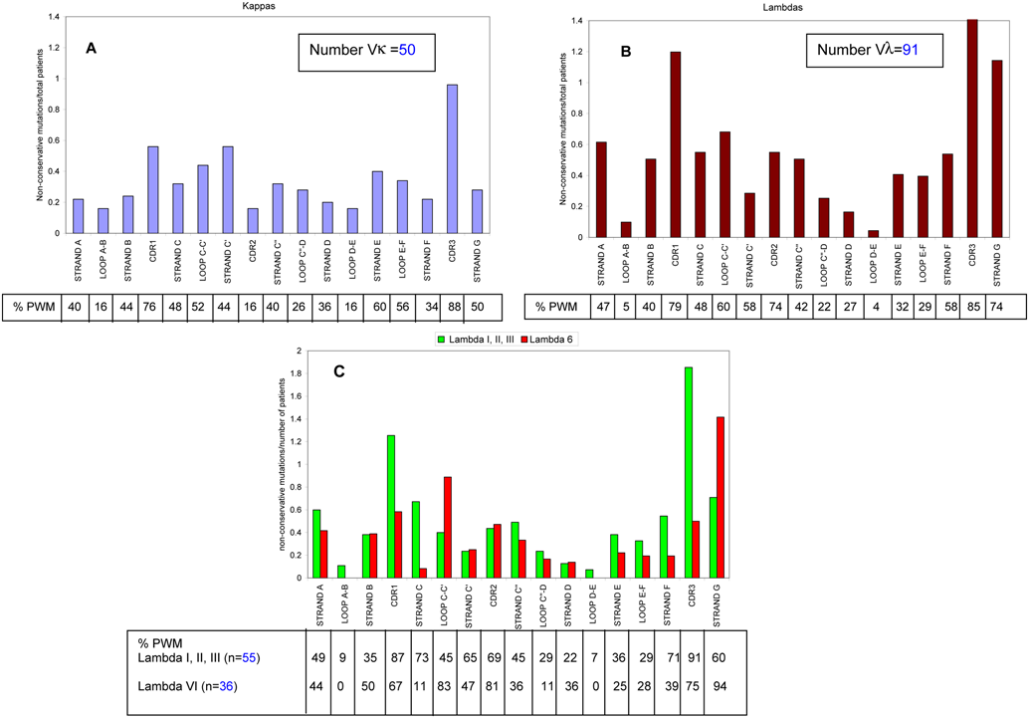
Non-conservative mutations over total number of patients for each secondary structure element for AL Vλ and Vκ proteins. The x-axis shows the different elements of secondary structure in the VL, while the y-axis gives the ratio of non-conservative mutations in each secondary structure element per total number of patients. The secondary structure boundaries used were based on the germline donor for each protein. Numbering is based on Kabat (http://vbase.mrc-cpe.cam.ac.uk/). A) Vκ sequences. B) Vλ sequences. C) Comparison of Vλ VI and other lambdas (Vλ I, II and III). The percentage of patients with mutations (% PWM) in each secondary structure element is listed per group.

The majority of the AL Vλ sequences in this study had a ratio of non-conservative over total mutation between 0.6–0.79, while the majority of AL Vκ sequences had a ratio between 0.4–0.59 (Table 1, Figure S10). Further analysis was performed comparing the proportion of conservative versus non-conservative mutations in all of our sequence groups (Table 2). Our data indicates that AL Vλ sequences have the widest range of non-conservative and total number mutations compared to all of the other sequence groups. When the number of non-conservative mutations per secondary structure over total number of patients for each germline group was calculated, no distinct pattern could be discerned between Vκ and Vλ, although the Vκ sequences showed a lower number of non-conservative mutations compared to Vλ sequences throughout the VL structure (Figure 2). The CDR regions accumulated more non-conservative mutations than any other region, in particular both CDR1 and CDR3 for AL Vκ and Vλ.

**Table 1:** Comparison of range of the fraction of non-conservative mutations/ total for all AL Vλ,Vκ, Mayo, Comenzo and Prokaeva Vλ VI, and all Vλ VI proteins.

Range fraction non conservative mutations/total	Total Vλ proteins	Total Vκ proteins	Mayo Vλ VI proteins	Comenzo Vλ VI	Prokeva Vλ VI	Total Vλ VI
0.8–1.0	11	10	3	1	1	5
0.6–0.79	47	15	6	7	5	18
0.4–0.59	32	17	3	8	2	13
0.2–0.39	0	8	0	0	0	0
0.0–0.19	1	0	0	0	0	0

The fraction of non-conservative mutations over total mutations were calculated for each patient and then classified according to their range.

**Table 2:** Comparison of the ranges of non-conservative of mutations and total number of mutations for all the protein sequences.

Mutations	AL-Vκ	AL-Vλ	Normal- Vκ	Normal Vλ	Multiple Myeloma
Non-Conservative Mutations	2–15	3–30	0–22	2–15	0–13
Total Mutations	3–18	4–38	1–32	2–17	2–18

The ranges of total mutations as well as the non-conservative mutations were calculated for each protein group including: AL-Vκ, AL-Vλ, Normal Vκ, Normal Vλ, and Multiple Myeloma.

**Figure 3:**
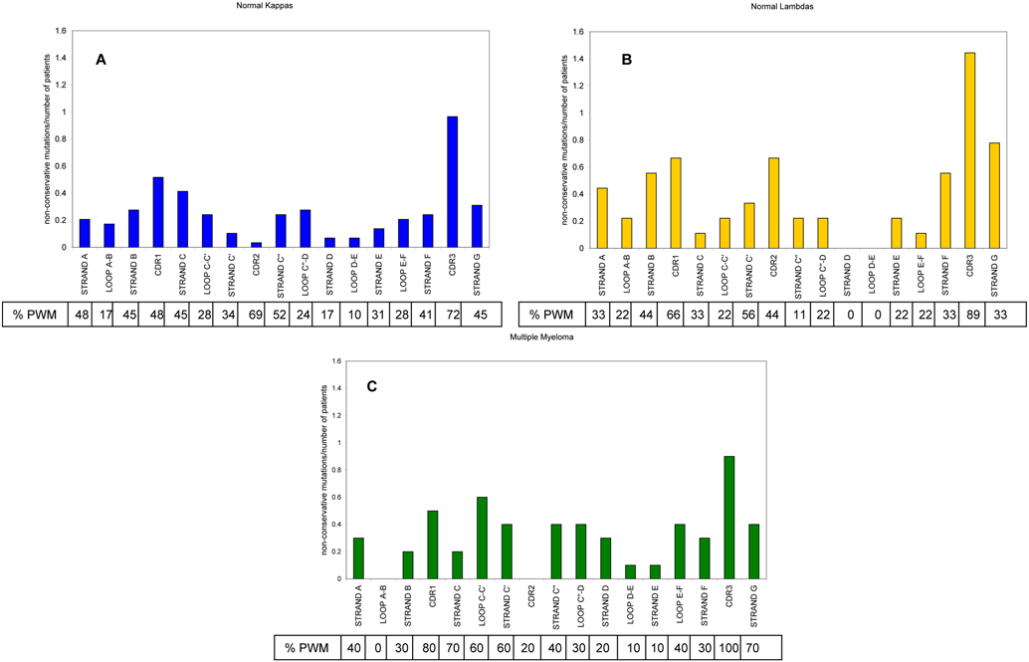
Non-conservative mutations over total number of patients for each secondary structure element for normal control Vλ, Vκ, and multiple myeloma control proteins. The x-axis shows the different elements of secondary structure in the VL, while the y-axis gives the ratio of non-conservative mutations in each secondary structure element per total number of sequences. The secondary structure boundaries used were based on the germline donor for each protein. Numbering is based on Kabat (http://vbase.mrc-cpe.cam.ac.uk/). A) Vκ normal control sequences. B) Vλ normal control sequences. C) Multiple Myeloma control sequences (combined Vλ and Vκ). The percentage of sequences with mutations (% PWM) in each secondary structure element is listed per group.

**Figure 4:**
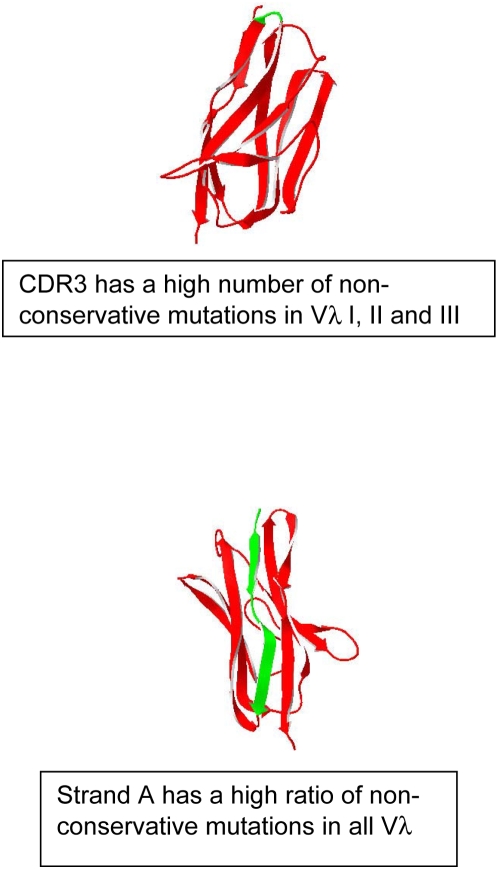
Structural models showing the common locations of non-conservative mutations in ALVλ proteins in our study. Protein models were based on the crystal structure for Vλ VI germline (2CDO.pdb). The β-strands in the structure are shown as red ribbons; mutation regions discussed in the captions are shown in green.

Because Vλ VI sequences are virtually always found in amyloid producing clones, the ratio of non-conservative mutations over total number of patients for Vλ VI versus VλI, II and III was analyzed (Figure 2C). The overall pattern for the Vλ I, II, and III proteins follows the same trends as the total Vλ group of sequences in Figure 2B. The Multiple Myeloma sequences follow the trend of the normal Vκ for the most part (Figure 3 and Figure S11). The Vλ VI proteins accumulate non-conservative mutations in loop C–C′ (part of CDR2) with 83% of patients with mutations in this region and presenting an absence of non-conservative mutations in loop A–B and loop D–E (structural representation of differences in Vλ is shown in Figure 4). High numbers of non-conservative mutations are found in β-strands A and G in all Vλ proteins; β-strand G has more non-conservative mutations in Vλ VI sequences. All of the control sequences show comparable levels of non-conservative mutations among each other.

Comparison of normal and AL sequences showed some interesting trends. Both the differences in the total number of mutations as well as the number of non-conservative mutations between normal sequences and AL Vκ are significant for loop C–C′ (p<0.0302 for total and p<0.0793 for non-conservative) and β-strand C′ (p<0.012 for total and p<0.006 for non-conservative). In the case of Vλ, the difference in the total number of mutations between normal and AL Vλ sequences for loop C–C′ is significant (p<0.0432) and so is the number of non-conservative mutations in β-strand C (p<0.108). We were interested in determining if Multiple Myeloma sequences would have significant differences in the location of non-conservative mutations compared to AL sequences. Multiple Myeloma is a plasma cell hematologic malignancy (as AL) but does not present amyloid deposits. In addition, Multiple Myeloma proteins have been used as non-amyloidogenic controls in biophysical studies [9], [10]. We found a significant difference in the total number of mutations between Multiple Myeloma and AL Vκ sequences in loop C–C′ (p<0.0919).

**Figure 5:**
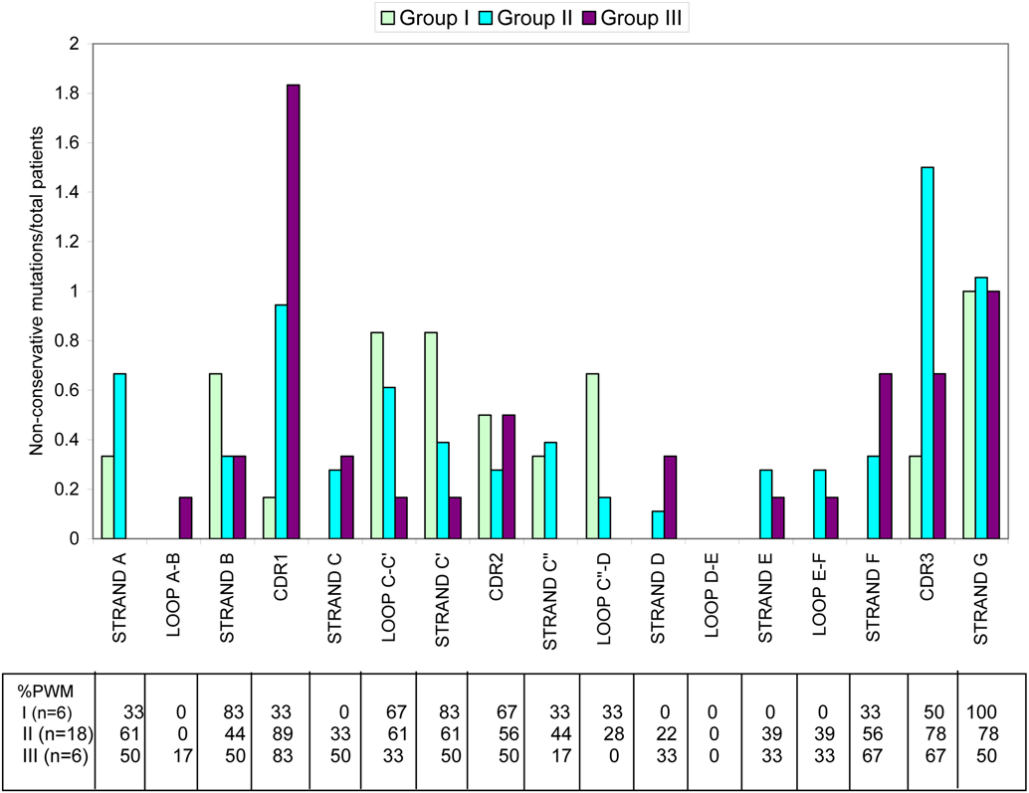
Comparison of non conservative mutations per total number of patients for low (I), medium (II) and high (III) iFLC levels in selected Mayo proteins per secondary structure. iFLC levels have been shown to be a good clinical parameter to follow disease progression [15], [18]. The sequences were gathered into three groups based on their iFLC levels at the time of diagnosis. Whenever there is no data shown for a particular group/secondary structure element, the value is zero. The secondary structure boundaries used were based on the germline donor for each protein. Numbering is based on Kabat (http://vbase.mrc-cpe.cam.ac.uk/). The %PWM in each secondary structure element is listed per group.

To test whether mutational patterns correlate with clinical parameters, sequences from 30 Mayo patients, who had serum free light chain (FLC) levels measured were further studied. Patients were divided into three groups based on the concentration of the involved serum FLC (iFLC) at the time of diagnosis [15]. Group I corresponded to low levels of iFLC (less than 10 mg/dL, including some patients with normal levels of iFLC, see methods section for the specific range); group II had intermediate levels (between 10.1–100 mg/dL) and group III had high levels of iFLC (above 100 mg/dL). A statistically significant difference in the number of non-conservative mutations was found among groups in β-strand A (p<0.03) and β-strand F (p<0.1). Group I and II present a large number of non-conservative mutations in β-strand A while group III had a high number of mutations in β-strand F. The only common region for absence of non-conservative mutations in all groups is loop D–E (Figure 5). The mutations in β-strand A for group I and II occur for the most part in amino acid positions pointing towards the surface of the protein. There is only one example where the mutation occurs in position 4 and the side chain points towards the protein core.

**Figure 6:**
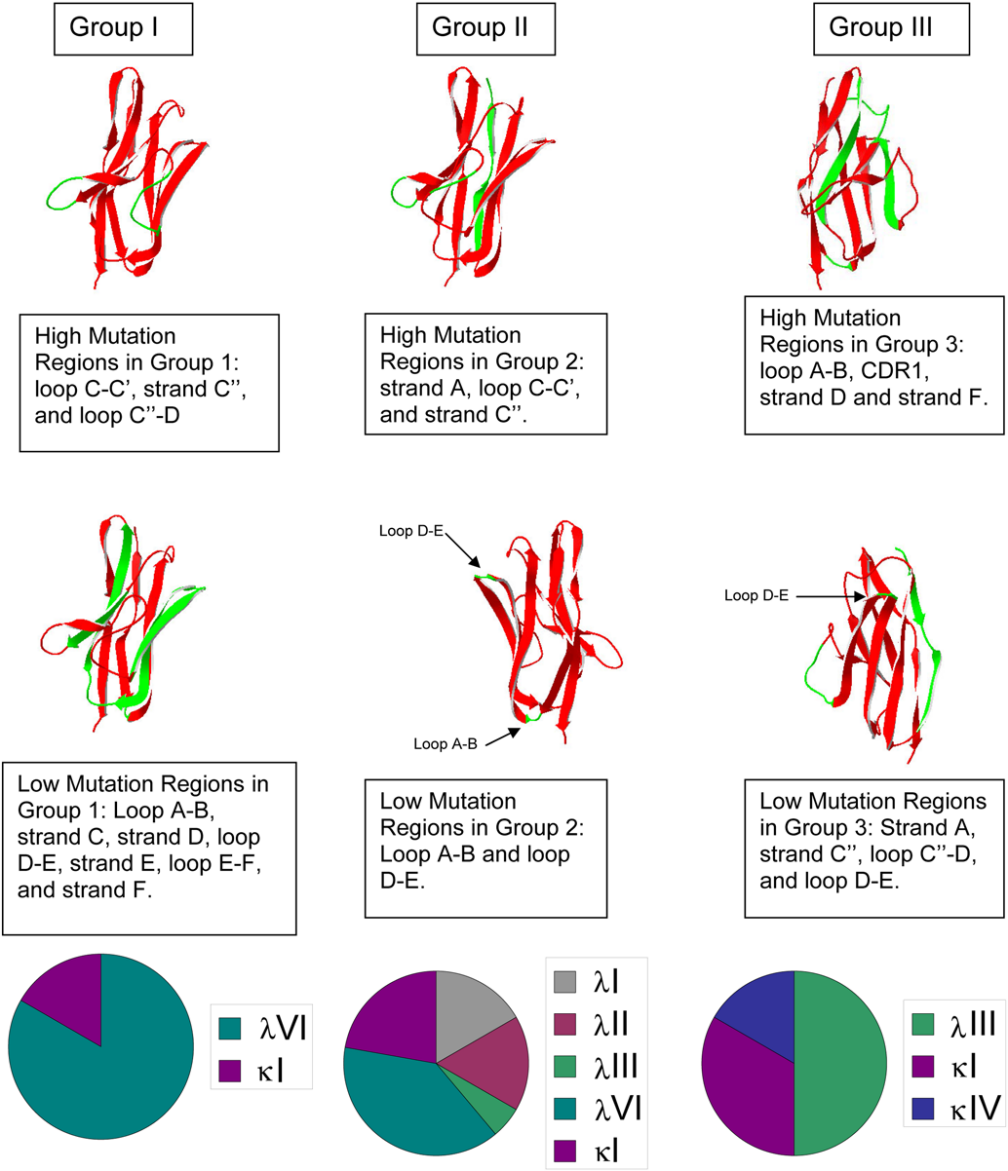
Structural models showing regions of high and low non-conservative mutation accumulation in iFLC level groups. Protein models were based on the crystal structure for Vλ VI germline (2CDO.pdb). The β-strands in the structure are shown as red ribbons; mutation regions discussed in the captions are shown in green.

There are many other regions that present unique patterns of high or low ratios of non-conservative mutations among the groups, but these regions do not attain statistical significance due to the small percentage of patients in each group with mutations in a given region (Figure 5). For example, a high number of non-conservative mutations were found for group I in β-strand B, loop C–C′ and β-strand C′. Group II and group III show a low number of mutations in these regions. The different germline types represented in these groups along with the regions of either high or low mutation accumulation in each group are shown in Figure 6.

A more detailed analysis of the types of non-conservative mutations seen in each protein was performed for group I. The 6 proteins in group I had a total of 49 mutations, 20 of which were considered conservative and 29 of which were considered non-conservative. Change in charge was the most common mutation (15 of 29) with gain of charge as the most frequent change of this group (9 of 15).

## Discussion

The results of our study showed that non-conservative mutations tend to accumulate in specific structural regions of the AL Vκ and Vλ sequences. Comparisons between normal and AL sequences identified discrete regions that have significantly higher numbers of mutations in the amyloidogenic sequences. The most interesting finding is that levels of iFLC at diagnosis corresponded with specific locations of non-conservative mutations in these sequences. AL is a protein misfolding disease with enormous mutational diversity. Efforts to understand the molecular determinants of amyloid formation for AL proteins could only be conducted in a large basis using sequence analysis of the subtypes in a separate fashion since κ and λ protein sequences do not share a large sequence identity. Stevens analyzed more than 100 κ1 AL LC family sequences from a larger sequence database, including 370 κ and λ LC entries [16]. He identified four structural risk factors for κ1 VL domains that may enhance the amyloidogenicity of LCs. These risk factors are: mutations in the isoleucine at position 27b; mutations in the amino acid at position 31 that change it to aspartic acid (both amino acids are located in the CDR1); mutations in Arginine 61 (located in strand D, part of β-hairpin DE), and the creation of glycosylation sites (Asparagine-X-Serine/Threonine) anywhere in the protein sequence. Our unique study compares VL sequences from AL patients by looking into the number of non-conservative mutations per secondary structure, extending the previous studies carried out so far by Stevens [16] to a new level. One important finding in our study is the fact that the number of mutations, total or non-conservative per protein, is not enough information to truly begin to understand the role of mutations in AL since Vκ normal controls present a wider range of non-conservative mutations compared to AL Vκ sequences (Table 2). We believe that it is essential to determine the number of non-conservative mutations over total number of patients per secondary structure in specific patient groups in order to extract useful information that could become relevant to the understanding of this disease. Our results also show that even though most mutations among AL proteins are non-conservative, some of the mutations present in their LCs are conservative and therefore may not affect the stability of the protein.

In addition, our results show that proteins grouped by iFLC levels clearly show distinct patterns in the location of non-conservative mutations. The statistical significance of the high number of non-conservative mutations in β-strand A (high number of non-conservative mutations in group I and II, absence of non-conservative mutations in group III) suggests that this region may play an important role in amyloidogenesis and is in agreement with studies from the Solomon group describing β-strand A as part of the cryptic epitope of VL for a monoclonal antibody against AL fibrils [17]. It is thought that over-expression of an amyloidogenic protein may increase the rate of amyloid formation and therefore will cause disease progression. The difference in the pattern of non-conservative mutations at different levels of iFLC suggests that non-conservative mutations in key areas of the immunoglobulin light chain may affect the rate of amyloidogenesis of the protein. It suggests the possibility that patients can have different amyloid formation rates despite similar light chain synthesis rate. Proteins with mutations identified in the group I patients may have the highest amyloid formation rates. As it has been published before [18], high iFLC levels may be associated with more advanced disease. Our results are the first indication that patients with low iFLC, which may appear to be at a lower risk for advanced disease, may be susceptible for amyloid formation because of the location of mutations in their proteins.

High numbers of non-conservative mutations in β-strand A were also observed in all Vλ. While it may appear that group I and II may be reflecting this same trend from their Vλ composition, non-conservative mutations have been observed in β-strand A in proteins from group II that are not Vλ.

Interestingly, some secondary structure elements completely lacked non-conservative mutations, such as β-strand F for group I. One way to explain this result is the fact that non-conservative mutations may matter more in certain secondary structure elements than others, thus presence or absence of non conservative mutations in certain types of proteins may not be relevant.

Significant differences were found between normals and AL Vκ and Vλ proteins in loop C–C′. This is an interesting finding since we have recently published crystallographic studies in which we propose that loss of interactions within loop C–C′ (also called the Proline-40 loop) could be involved in the initial conformational changes leading to amyloid formation [19].

The unique pattern of non conservative mutations found in this study may have future implications in the treatment of AL patients given that knowledge of the position of non-conservative mutations could potentially be used as a marker for disease progression and response to therapy.

In conclusion, the study of the position of non-conservative mutations could not only help us understand the molecular mechanisms in amyloid formation for AL, but it has the potential to become a new prediction tool for AL disease progression and response.

## Materials and Methods

### Ethics statement

The study was carried out under an institutional review board (IRB)-approved protocol and followed the Helsinki guidelines for research of human subjects.

### Sequence sources

Available sequences were selected from the Abraham, Comenzo, Prokaeva, Arendt, Wally, and Sikkink studies in an unbiased way. The only requirements were that the cDNA sequence was unambiguous (did not contain unassigned ‘N’ nucleotides) and complete (included the entire VL domain starting from the FR1 down to the FR4). Sequences obtained from Abraham, Sikkink, and Arendt studies will be referred to as Mayo sequences, those obtained from Comenzo and Wally will be referred to as Comenzo sequences in this article. The Prokaeva group comes from Prokaeva et al 2007 [8]. We sequenced two additional proteins in order to incorporate more Vλ VI sequences that were underrepresented from the Mayo cohort of sequences. A total of 55 Vλ I, II and III sequences were used in this study, which were obtained from Abraham, Comenzo, Prokaeva and Sikkink publications. The GenBank accession numbers for the Vλ I, II and III from Abraham et al 2003 [7] include: AF490938, AF490940, AF490941, AF490944, AF490945, AF490949, AF490952, AF490953, AF490955, AF490958, AF490960, and AF490961. AY730911 and AY730938 are from Abraham et al 2007 [20]. The GenBank accession numbers for Vλ I, II and III from Comenzo et al 1998 publication [21] include: AF124170, AF124163, AF124165, AF124164, AF124172, AF124176, AF124173, AF124171, AF124175, AF124174, and AF124186. We included the following sequences from Comenzo et al 1999 [22]: AF054641, AF054640, AF115347, AF054638, AF115350, AF115349, AF115354, and AF054647. AF320832, AF320833, and AF320834 are from Comenzo et al 2001 [6]. The sequences: ABU90545, ABU90717, ABU90728, ABU90727, ABU90724, ABU90719, ABU90703, ABU90725, ABU90704, ABU90701, ABU90548, ABU90705, ABU90553, ABU90552, ABU90550, ABU90732 and ABU90723 were from Prokaeva et al. 2007 [8]. The GenBank accession numbers for Vλ I, II and III from Sikkink et al [23] include: DQ240234 and DQ240235.

There were also 50 Vκ sequences used for comparison in this study, which were obtained from Abraham, Comenzo, Prokaeva, Sikkink and Wally publications. The GenBank accession numbers for the Vκ sequences from Abraham et al 2003 [7] include: AF490909, AF490910, AF490912, AF490913, AF490916, AF490920, AF490921, AF490925, AF490929, AF490908, AF490917, AF490922, AF490907, AF490911, AF490924, and AF490937. We included AY701640 from Abraham et al 2007 [20]. The sequences ABU90544, ABU90674, ABU90662, ABU90652, ABU90647, ABU90625, ABU90604, ABU90716, ABU90653, ABU90600, ABU90599, ABU90712, ABU90644, ABU90636, ABU90633, ABU90602, ABU90598, ABU90713, ABU90663, ABU90648, ABU90646, and ABU90637 are from Prokaeva et al. 2007 [8].The sequence DQ240237 from Sikkink et al 2008[23] was initially deposited when the patient had multiple myeloma. The patient has since evolved to AL. The Genbank accession numbers for the Vκ sequences from Comenzo et al 1998 [21] include: AF124197, AF361758, and AF124193, AF1156361, AF054662, AF054661, AF054658, and AF054656 from Comenzo et al 1999 [22] and AF320835 from Comenzo et al 2001[6]. We included AF113887 from Wally et al 1999 [24].

A total of 36 Vλ VI were used in this study. The Vλ VI sequences obtained for the study were from Abraham, Comenzo, Prokaeva, and Arendt publications. Two of the cDNA sequences for Vλ VI were done in our laboratory. The Gen Bank accession numbers for the Vλ VI from Comenzo et al 1998 [21] include: AF124189, AF124184, AF124181, AF124190, AF124187, and AF124185; sequences from Comenzo et al 1999 [22] include: AF115360, AF115358, AF115357, AF054653, AF054651, and AF054649; and the sequences AF320840, AF320839, AF320838, and AF320837 were from Comenzo et al 2001[6]. The Vλ VI sequences obtained from Abraham et al 2003 [7] include: AF490966, AF490967 and AF490968. The Vλ VI sequences obtained from Abraham but not yet published include: EF710984, EF710878, EF711037, EF710969, EF710946, and AY793337. The following sequences: ABU90551, ABU90549, ABU90726, ABU90722, ABU90720, ABU90711, ABU90707, and ABU90699 were from Prokaeva et al 2007 [8]. FJ200244 was published by Arendt et al 2008 [25], and the Vλ VI sequenced in our laboratory for this study are: FJ172996 and FJ172997.

Normal control proteins were selected from the NCBI website: http://www.ncbi.nlm.nih.gov/sites/entrez?db=Protein&itool=toolbar by searching “human immunoglobulin light chain antibody NOT amyloidosis”. The Vκ sequences include: CAA66157, CAA66153, CAA59989, CAA59988, AAX14398, AAB49705, BAH03699, CAA39072, BAF64543, AAC02819, AAB41730, AAA20168, AAA20167, AAA20163, BAH03697, BAH03696, AAA20160, AAB49706, AAA20158, CAE54366, AAD29271, AAB41731, AAA20165, CAE54365, CAE54364, CAE54363, CAE54362, CAE54361 and AAD14088. The Vλ sequences include: AAA59018, AAC08342, AAC06030, CAA65054, BAA19564, BAA19562, CAA36351, AAC08338 and AAA75556.

Some Multiple Myeloma protein sequences were also used for comparison. These sequences include: 1CD0.pdb (Multiple Myeloma protein JTO, published in [9]), 1REI.pdb (protein REI), protein GAL as published by Kim et al. 2000 [10], 1lve.pdb (LEN), AY701647, AY701035, AY730974, AY701728, AY701699, and DQ240236.

Some of the AL cDNA samples were re-sequenced in our laboratory because the sequences were not complete in GenBank and we had access to the cDNA. The GenBank accession numbers for these sequences are: AF490938, AF490960, AF490911, AF490929, AF490937, and AF490968. These sequences have been updated in GenBank.

### VL cDNA sequencing

For the sequences determined in our laboratory, bone marrow (BM) aspirates were collected previously as described in Abraham et al 2003 [7] from patients with biopsy-proven AL who were seen in the Hematology Division at the Mayo Clinic. Briefly, the marrow preparations were layered on Ficoll Paque to remove red blood cells, and the mononuclear cells were washed and frozen at -80°C. Total RNA was extracted from the cells using Trizol Reagent. The RNA was then used for cDNA preparation using Superscript reverse transcriptase. Since we had at least partial cDNA sequences for these patients, 5′ primers were designed to target the specific leader sequence for the germline of these patients along with a 3′ constant region primer for λ or κ. The degenerate primers used in this study were previously reported by Abraham et al. 2003 [7] based on the primers initially reported by Welschof et al. 1995 [26]. Most of the specific primers used in this study were published by Abraham et al. 2003 [7]. In addition, we used the following primers from Comenzo et al. 2001 [6]:

VL3 3r ATG GCA TGG ATC CCT CTC TTC

VL6 6a ATG GCC TGG GCT CCA CTA CTT

These additional primers were designed and used in this study:

VL1 1e ATG GCC TGG TCT CCT CTC CTC

VK2-A17 ATG AGG CTC CCT GCT CAG CTC CTG

VK1-L1 ATG GAC ATG AGA GTC CTC GCT CAG

VKIV B3 GGA TCT CTG GTG CCT ACG GGG

The appropriate DNA band was cut and purified using the Qiagen QIAquick gel extraction kit. The PCR product was cloned into pCR2.1 TOPO using the TOPO TA cloning kit from Invitrogen. Twelve of the resulting clones were sequenced with forward primers at the Mayo Molecular Biology Sequencing Core Facility. The clonal VL gene was determined if one gene was clearly overrepresented in each patient and the protein sequences were identical in at least five PCR products. The clonal nature of the Comenzo sequences was determined in a similar manner. In the case of the Prokaeva sequences, the clonal sequence was determined by the identity of at least 50% of 6–9 independently cloned and sequenced products.

DNA sequences obtained were analyzed using DNAPLOT from the VBase website (http://vbase.mrc-cpe.cam.ac.uk/). This database uses all known human light chain germline sequences to assign germline donors based on comparison of the sequences for the most nucleotide homology.

### Structural Characterization of VL Sequences

Once a germline donor was assigned, the sequence of each protein was modeled on known light chain structures using the Swiss-PdbViewer 3.7 from the website http://www.expasy.org/spdbv/. Crystal structures have been reported for κI (O18/O8) (1B6D.pdb), κII (2AI0.pdb), κ IV (1LVE.pdb), λ2b2 (1JVK.pdb) and λVI (2CD0.pdb) proteins, so we aligned the remaining germline sequences with those germline sequences represented in the crystal structures using BLAST2 (http://www.ncbi.nlm.nih.gov/blast/bl2seq/wblast2.cgi) to find the best match for structural modeling. The κI (O18/O8) crystal structure was used for all kI and kIII sequences; kII and kIV protein sequences have their own crystal structure representatives. The Vλ VI crystal structure was used for the Vλ III, Vλ VI, VII, and IX sequences. The Vλ II (2b2) crystal structure was used for all Vλ I and Vλ II sequences. The β-strands and loops were assigned using the secondary structure information from spdb viewer, but the numbering used was according to Kabat from the VBase website (http://vbase.mrc-cpe.cam.ac.uk/). Each patient protein sequence was aligned with their own germline sequence, mutations were identified and highlighted and then examined to determine the secondary structure location of the mutations. The AL Vλ proteins were divided to compare the Vλ VI proteins to all the other Vλ proteins (Vλ I, II, and III). Conservative mutations were those that had similar chemistry (polar to polar, similar charge, and similar size). We also considered a mutation from valine, leucine, and isoleucine to/from phenylalanine to be conservative based on the hydrophilicity measured using side chain analogues by Radzicka and Wolfenden [27]. Non-conservative mutations were those that resulted in a change in charge, change in hydrophobicity, change in side chain size, and inclusion/replacement of proline or glycine. We divided the number of non-conservative mutations found per secondary structure by the total number of patients in that specific group. The same pattern of high and low non-conservative mutations values were found when we multiplied the number of non-conservative mutations times the ratio of patients with non-conservative mutations over the corrected total number of patients in a given category. We also calculated the ratio of non-conservative mutation over total number of mutations, but this ratio did not represent the true distribution of patients among a specific group.

To test whether mutational position determines amyloidogenesis, AL patients with different levels of circulating serum free light chain at the time of diagnosis were identified. Serum free light chains were measured using the Freelite™ Serum Free Light Chain Assays (The Binding Site Inc., San Diego, CA). Normal range for κ is 0.33 to 1.94 mg/dL and 0.57 to 2.63 mg/dL for λ. Measurements were made with serum samples taken closest to the time of diagnosis. Patients were separated in groups based on the levels of the pathologic iFLC. The cutoffs for each group were assigned based on the logarithmic increments. The low level group had iFLC ≤10 mg/dL (including patients with iFLC within normal ranges listed above), 10.1 to 100 mg/dL in the intermediate group and >100 mg/dL for the high group.

## Supporting Information

Figure S1 Sequence alignment of AL VκI O18/O8, L1 and VK1106*01. All protein sequences were grouped based on the dominant clone identified in a given patient's bone marrow sample. For κ sequences, secondary structure was based on κ light chain protein models κI (1B6D.pdb), κII (2AI0.pdb) and κ IV (1LVE.pdb), using Swiss Protein Database Viewer. For Vλ proteins (I, II, III) and Vλ VI proteins secondary structure was based on Vλ protein model (1JVK.pdb) and (2CD0.pdb) using Swiss Protein Database Viewer, respectively. Numbering for the secondary structure was based on Kabat (http://vbase.mrc-cpe.cam.ac.uk/). Sequences are called according to their GenBank numbers. Bold sequences correspond to the germline donor sequence. Yellow highlights denote somatic mutations present in the sequences.
(0.95 MB TIF)

Figure S2 Sequence alignment of AL VκI L12, 012/02, L5, and VκIV B3. Structure determination and mutation analysis were done as described in Figure S1.
(0.75 MB TIF)

Figure S3 Sequence alignment of AL VλI 1c, 1b and 1e. Structure determination and mutation analysis were done as described in Figure S1.
(0.87 MB TIF)

Figure S4 Sequence alignment of AL VλII 2b2, 2a2, 2c, and VλIII 3r. Structure determination and mutation analysis were done as described in Figure S1.
(1.09 MB TIF)

Figure S5 Sequence alignment of AL VλVI 6a. Structure determination and mutation analysis were done as described in Figure S1.
(1.09 MB TIF)

Figure S6 Sequence alignment of Normal Control VκI L12, 012/02, L5, VκII A19, A1, VκIII A27 and L2. Structure determination and mutation analysis were done as described in Figure S1.
(0.81 MB TIF)

Figure S7 Sequence alignment of Normal Control VκIII L6 and L2 VκIV B3. Structure determination and mutation analysis were done as described in Figure S1.
(0.42 MB TIF)

Figure S8 Sequence alignment of Normal Control VλI 1c, 1b, VλII 2a2, 2e, VλIII 3h, VλVII 7b and VλIX 9a. Structure determination and mutation analysis were done as described in Figure S1.
(0.59 MB TIF)

Figure S9 Sequence alignment of Multiple Myeloma Control VλVI 6a, VλIII 3r, VλII 2a2, VκI 018/08, L12, VκII A19, and VκIV B3. Structure determination and mutation analysis were done as described in Figure S1.
(0.69 MB TIF)

Figure S10 The total mutations were counted for each AL patient and the fraction of those that were considered non-conservative graphed. The majority of the AL Lambda patients had a fraction of non-conservative mutations falling between 0.6 and 0.79, whether they were Lambda I, II and III or Lambda VI. The majority of AL Kappa patients had a fraction of non-conservative mutations falling between 0.4 and 0.59.
(0.78 MB TIF)

Figure S11 Comparison of the number of non-conservative mutations over total number of individuals between Multiple Myeloma, normal kappas and normal lambdas (Data from Figure 3).
(0.47 MB TIF)
