# Supplementary figures and images for: Mutations in Specific Structural Regions of Immunoglobulin Light Chains Are Associated with Free Light Chain Levels in Patients with AL Amyloidosis
Source: PLoS One. 2009 Apr 13;4(4):e5169. doi: 10.1371/journal.pone.0005169 (PMC2664898; doi:10.1371/journal.pone.0005169)

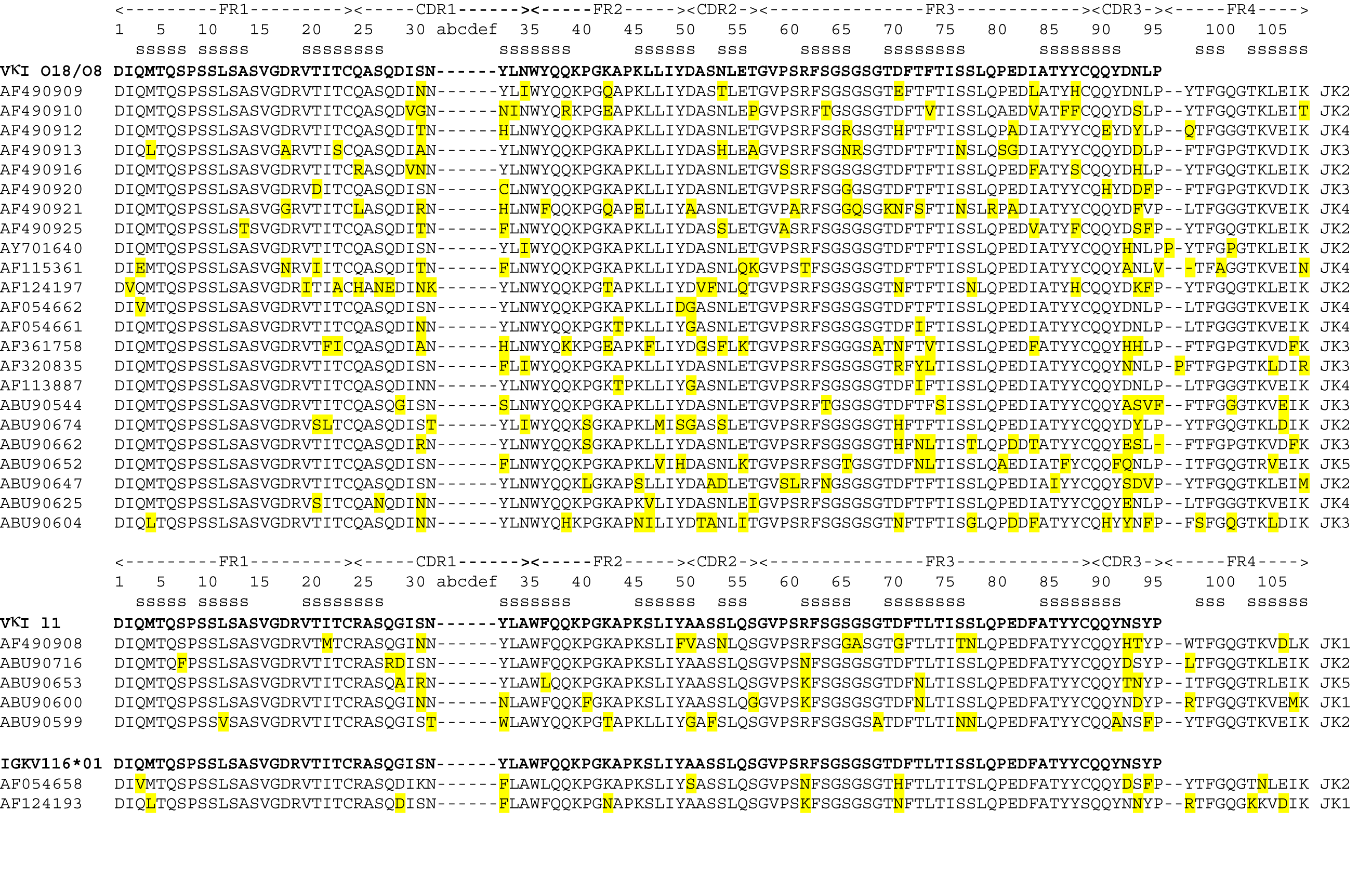

Supplement: Figure S1 — Sequence alignment of AL VκI O18/O8, L1 and VK1106*01. All protein sequences were grouped based on the dominant clone identified in a given patient's bone marrow sample. For κ sequences, secondary structure was based on κ light chain protein models κI (1B6D.pdb), κII (2AI0.pdb) and κ IV (1LVE.pdb), using Swiss Protein Database Viewer. For Vλ proteins (I, II, III) and Vλ VI proteins secondary structure was based on Vλ protein model (1JVK.pdb) and (2CD0.pdb) using Swiss Protein Database Viewer, respectively. Numbering for the secondary structure was based on Kabat (http://vbase.mrc-cpe.cam.ac.uk/). Sequences are called according to their GenBank numbers. Bold sequences correspond to the germline donor sequence. Yellow highlights denote somatic mutations present in the sequences. (0.95 MB TIF) [file pone.0005169.s001.tif]

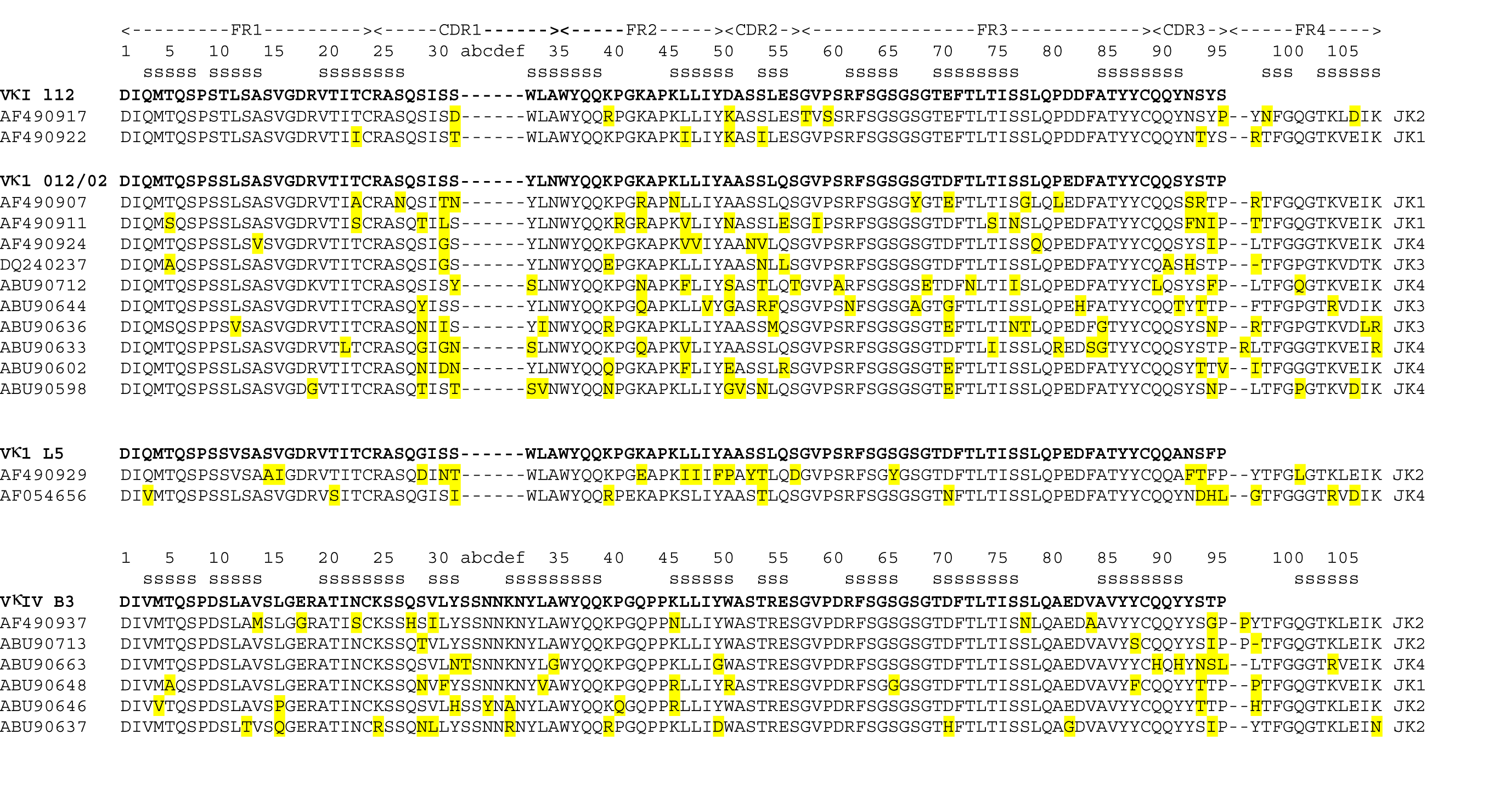

Supplement: Figure S2 — Sequence alignment of AL VκI L12, 012/02, L5, and VκIV B3. Structure determination and mutation analysis were done as described in Figure S1. (0.75 MB TIF) [file pone.0005169.s002.tif]

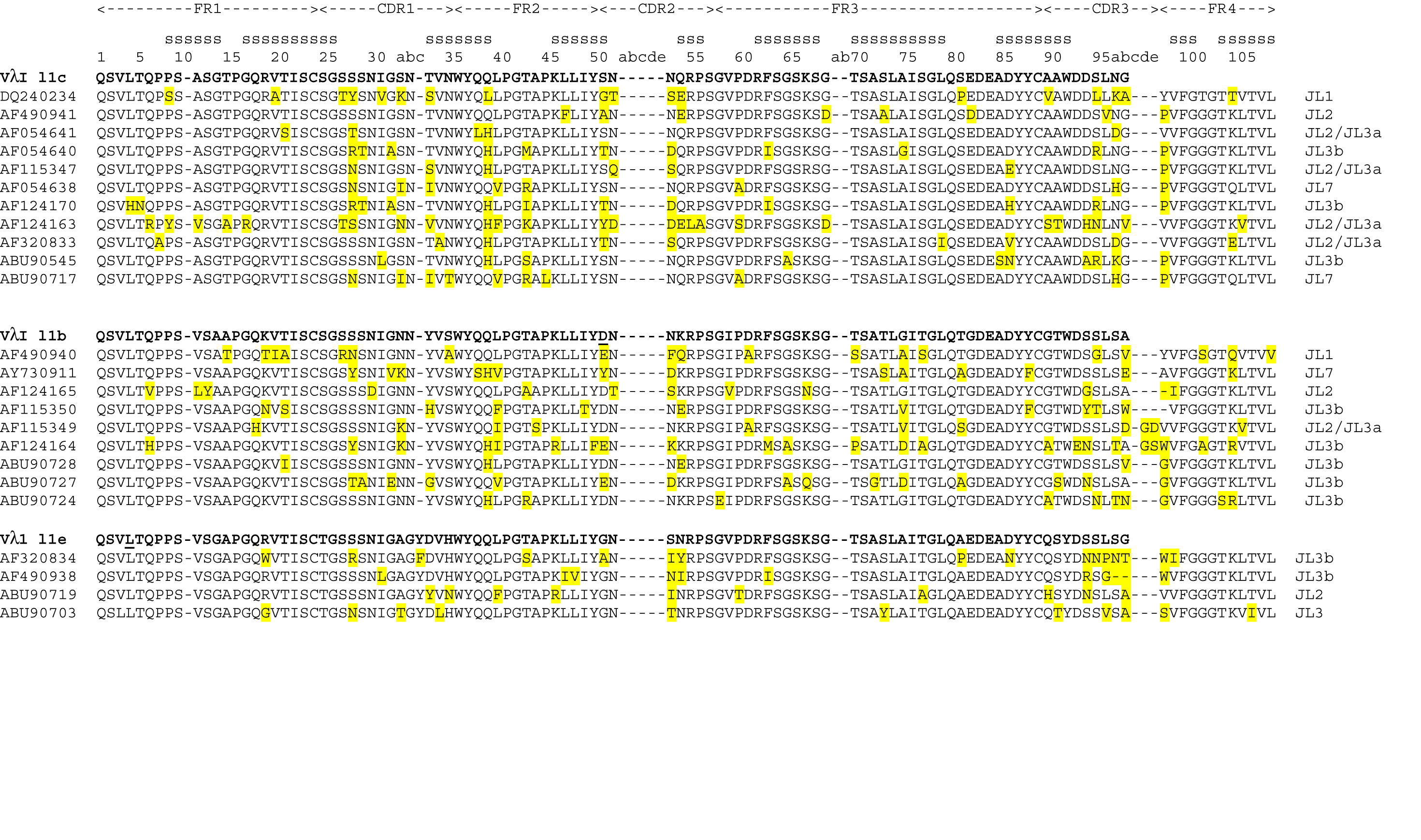

Supplement: Figure S3 — Sequence alignment of AL VλI 1c, 1b and 1e. Structure determination and mutation analysis were done as described in Figure S1. (0.87 MB TIF) [file pone.0005169.s003.tif]

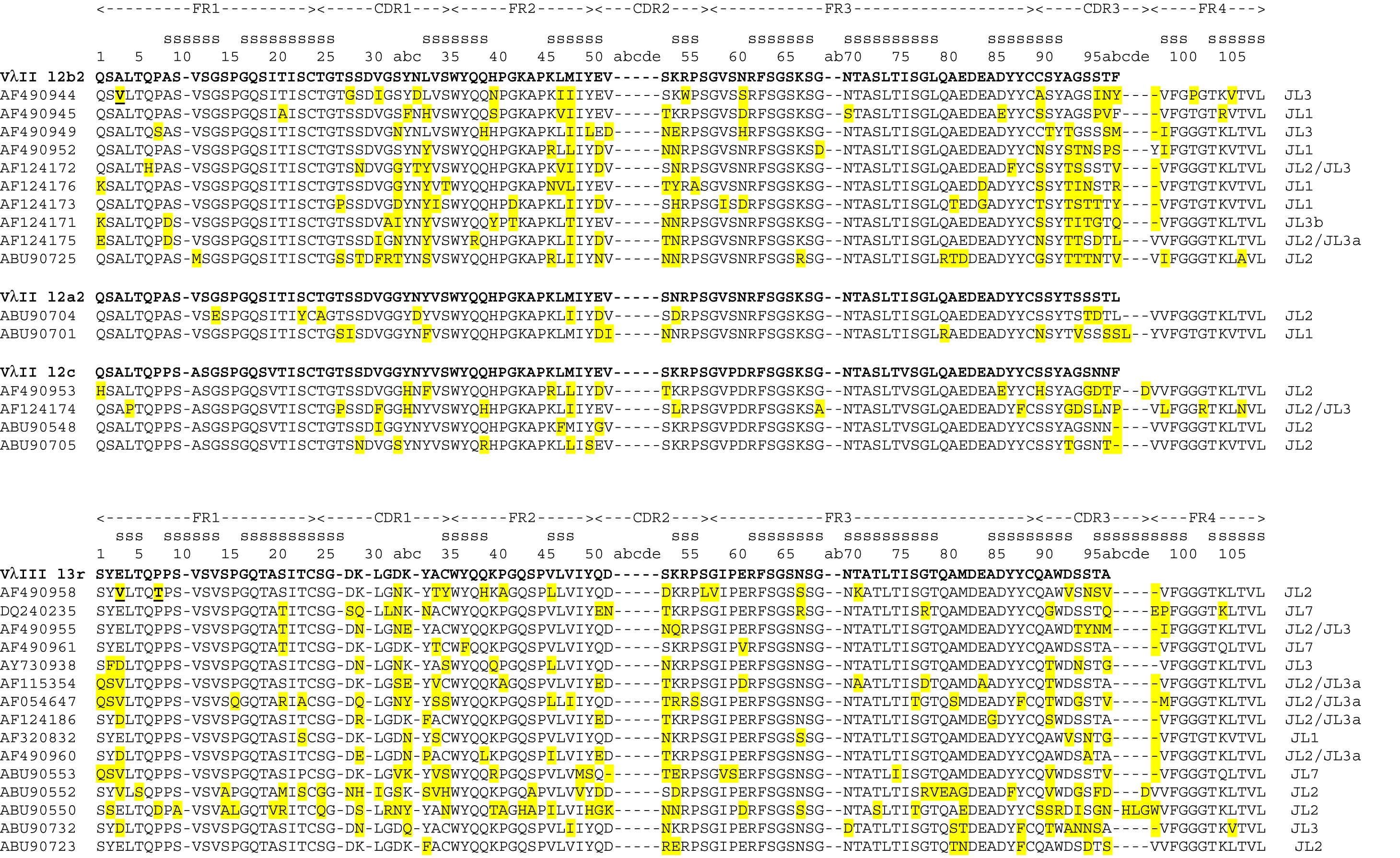

Supplement: Figure S4 — Sequence alignment of AL VλII 2b2, 2a2, 2c, and VλIII 3r. Structure determination and mutation analysis were done as described in Figure S1. (1.09 MB TIF) [file pone.0005169.s004.tif]

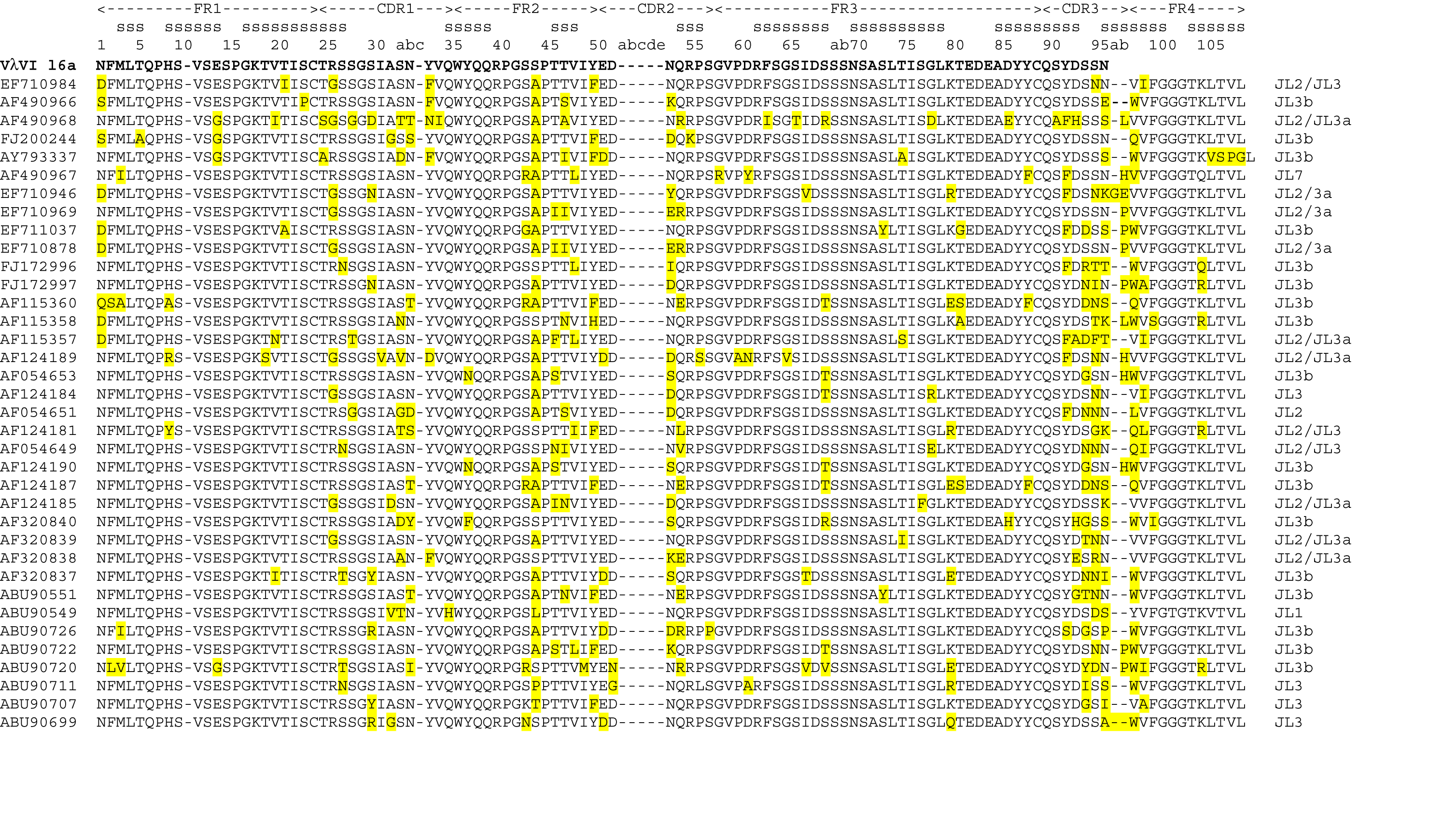

Supplement: Figure S5 — Sequence alignment of AL VλVI 6a. Structure determination and mutation analysis were done as described in Figure S1. (1.09 MB TIF) [file pone.0005169.s005.tif]

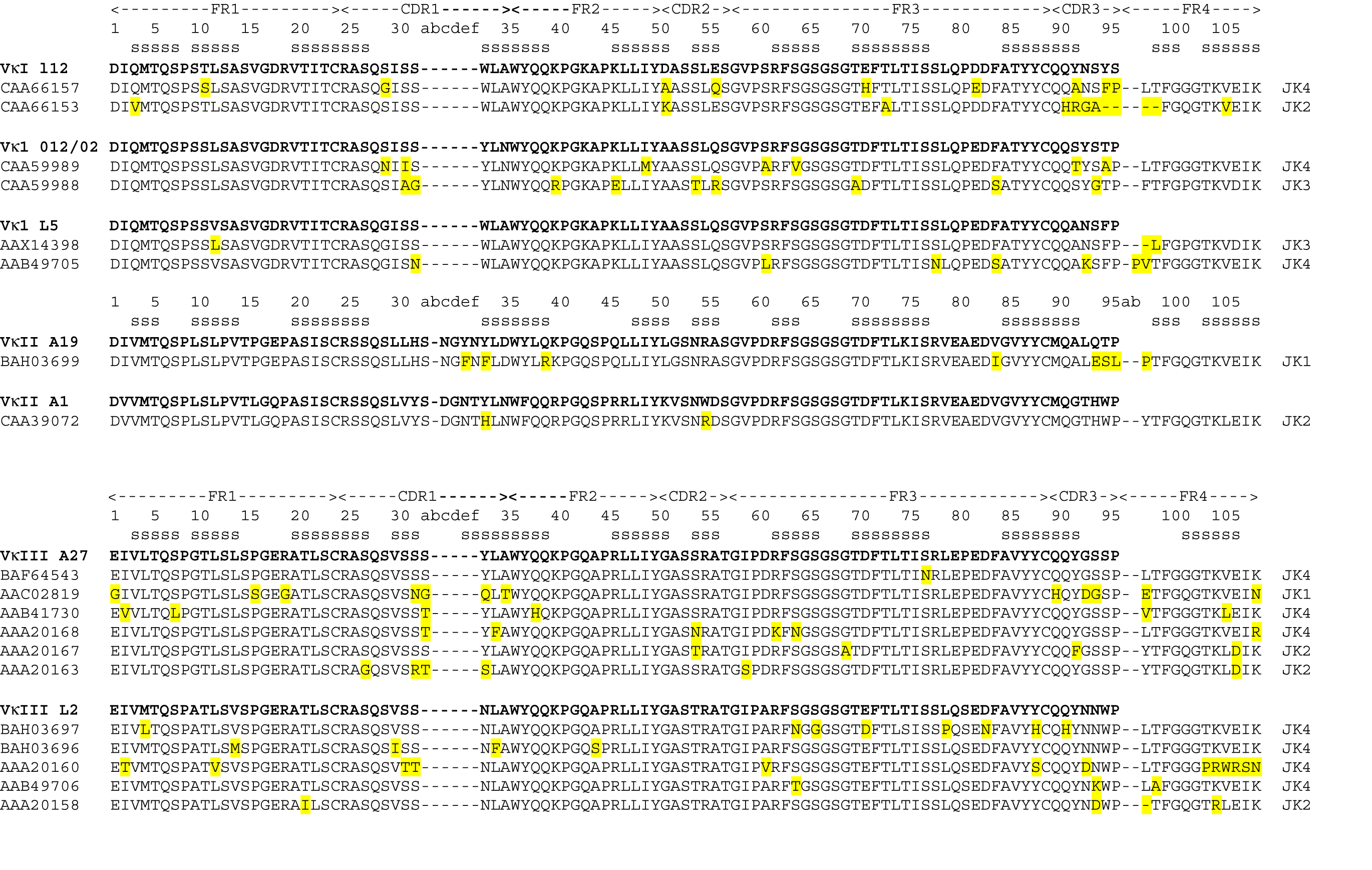

Supplement: Figure S6 — Sequence alignment of Normal Control VκI L12, 012/02, L5, VκII A19, A1, VκIII A27 and L2. Structure determination and mutation analysis were done as described in Figure S1. (0.81 MB TIF) [file pone.0005169.s006.tif]

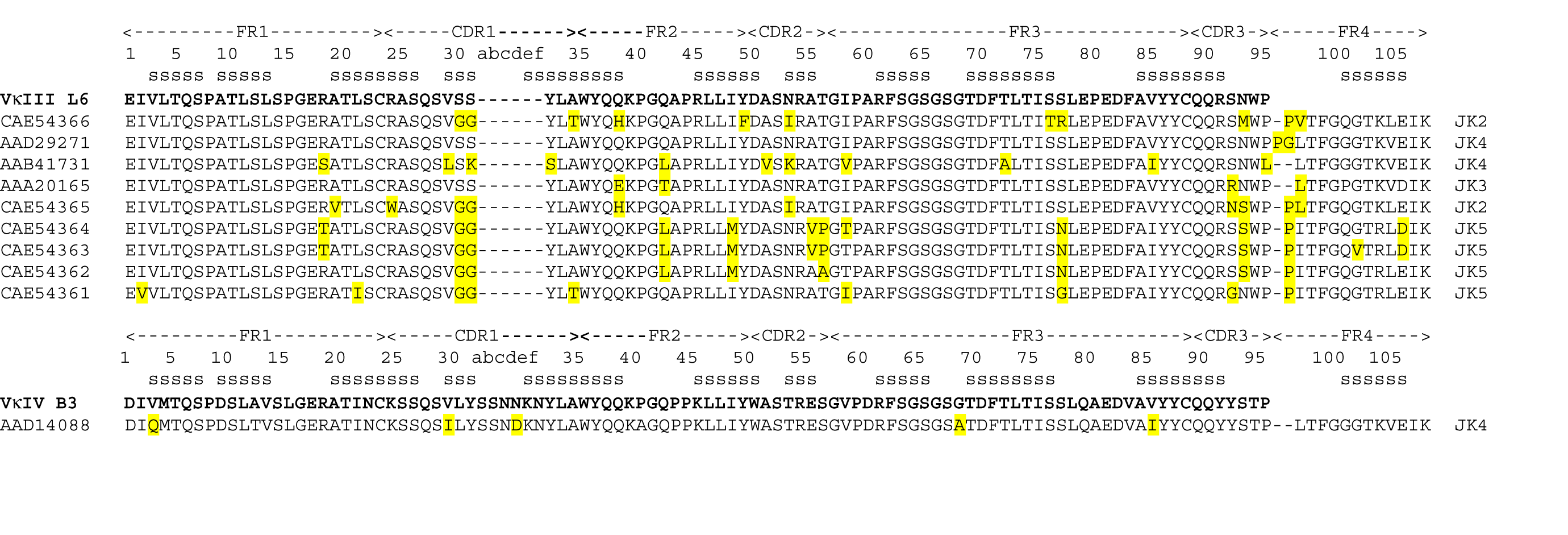

Supplement: Figure S7 — Sequence alignment of Normal Control VκIII L6 and L2 VκIV B3. Structure determination and mutation analysis were done as described in Figure S1. (0.42 MB TIF) [file pone.0005169.s007.tif]

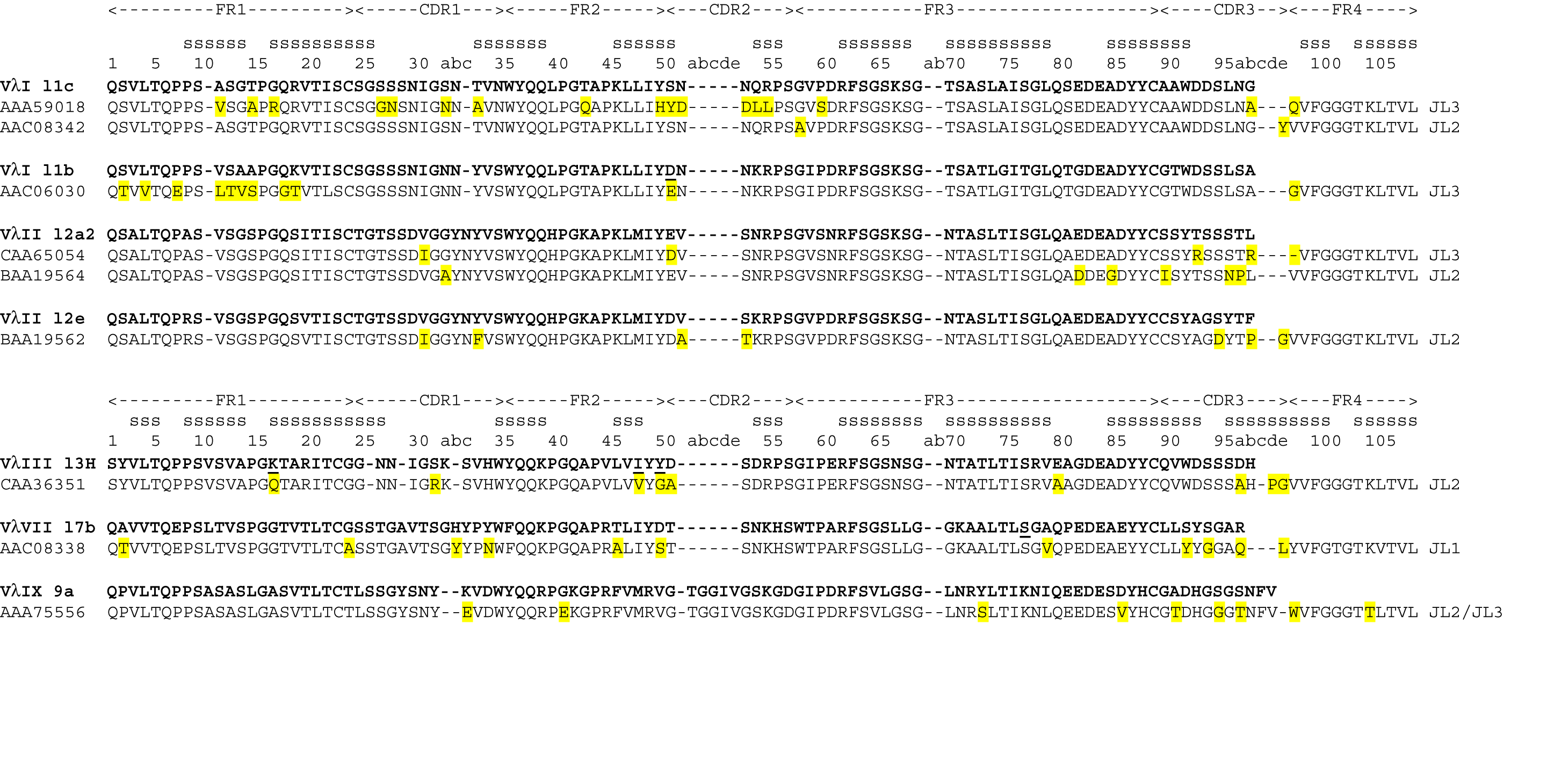

Supplement: Figure S8 — Sequence alignment of Normal Control VλI 1c, 1b, VλII 2a2, 2e, VλIII 3h, VλVII 7b and VλIX 9a. Structure determination and mutation analysis were done as described in Figure S1. (0.59 MB TIF) [file pone.0005169.s008.tif]

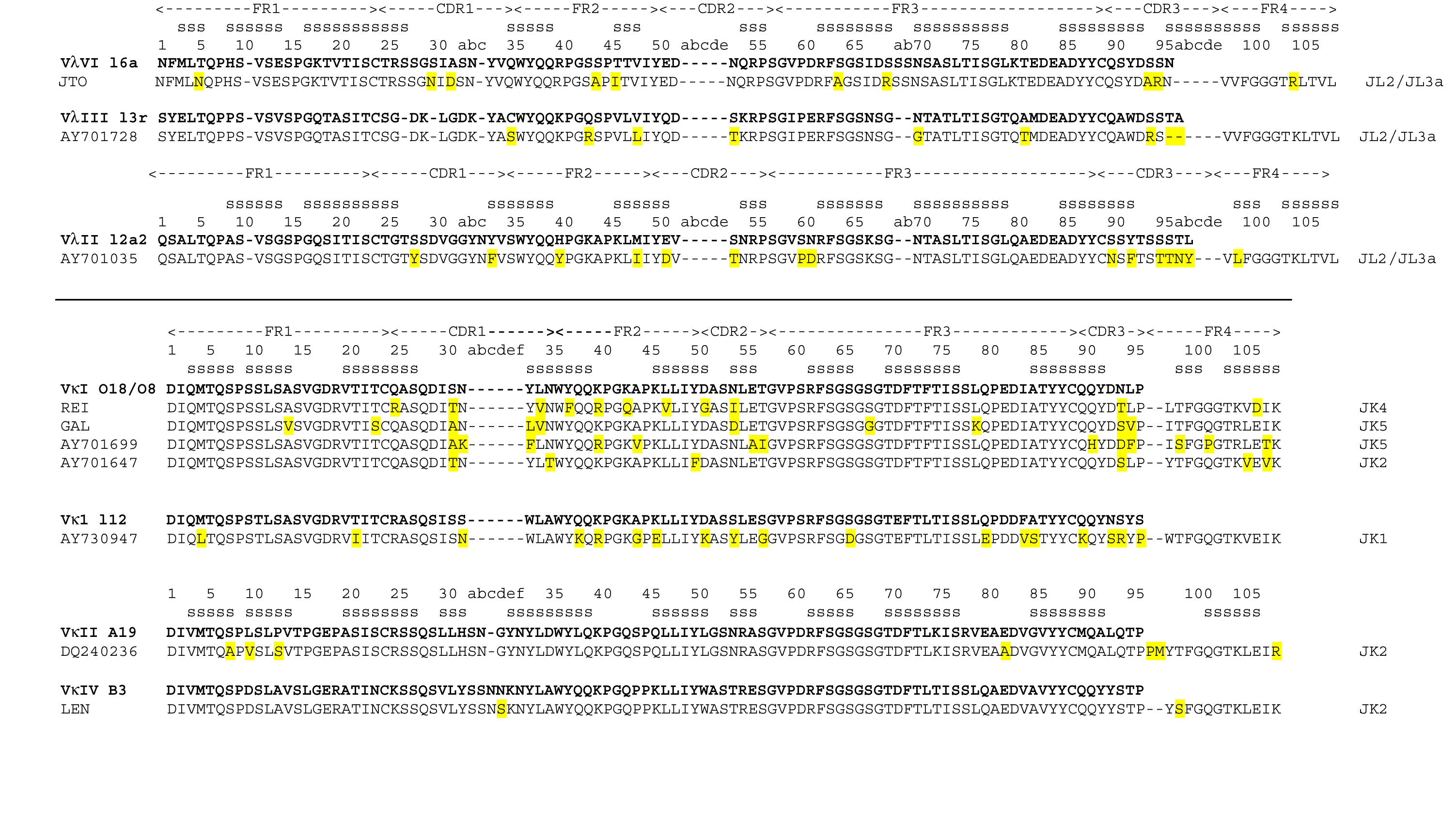

Supplement: Figure S9 — Sequence alignment of Multiple Myeloma Control VλVI 6a, VλIII 3r, VλII 2a2, VκI 018/08, L12, VκII A19, and VκIV B3. Structure determination and mutation analysis were done as described in Figure S1. (0.69 MB TIF) [file pone.0005169.s009.tif]

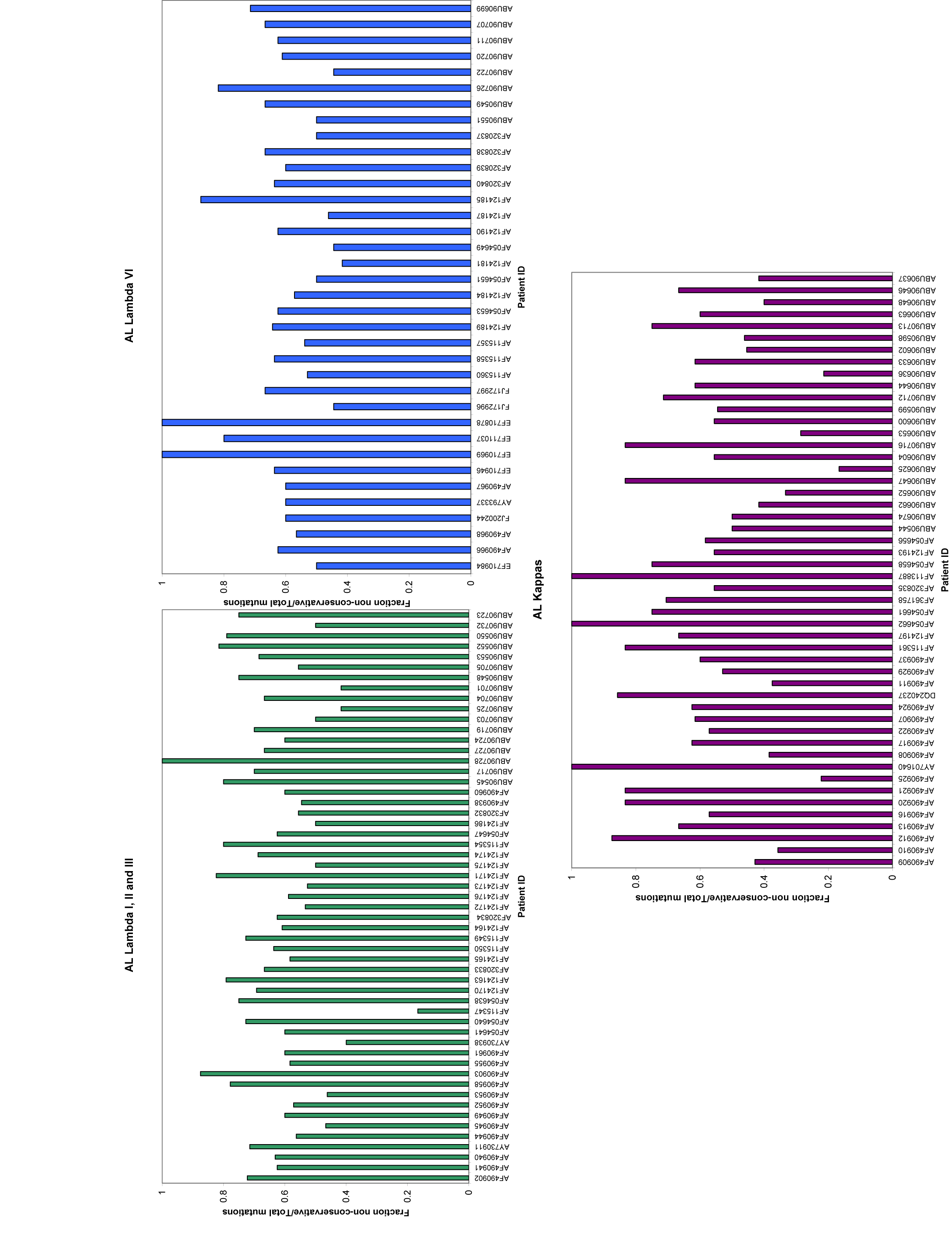

Supplement: Figure S10 — The total mutations were counted for each AL patient and the fraction of those that were considered non-conservative graphed. The majority of the AL Lambda patients had a fraction of non-conservative mutations falling between 0.6 and 0.79, whether they were Lambda I, II and III or Lambda VI. The majority of AL Kappa patients had a fraction of non-conservative mutations falling between 0.4 and 0.59. (0.78 MB TIF) [file pone.0005169.s010.tif]

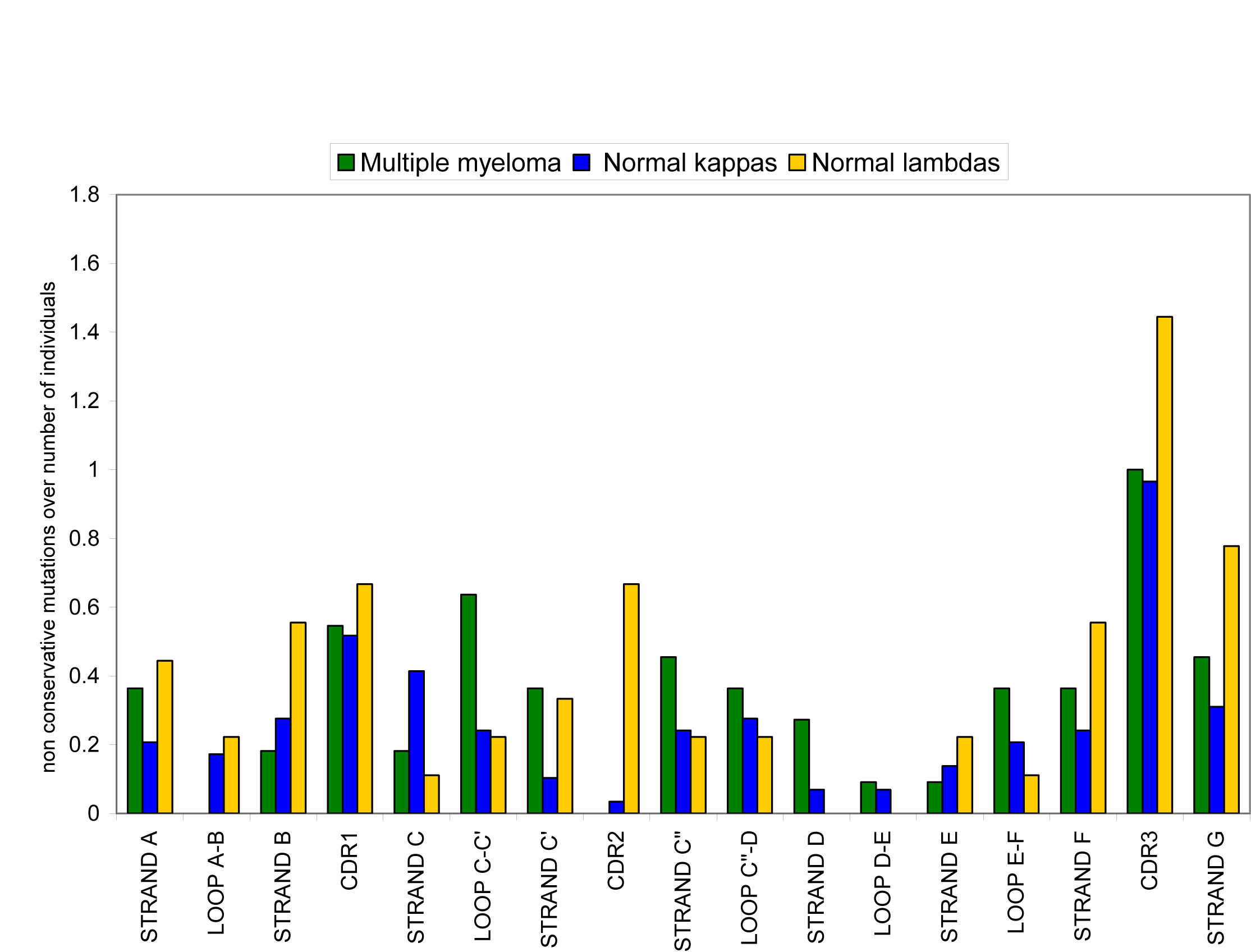

Supplement: Figure S11 — Comparison of the number of non-conservative mutations over total number of individuals between Multiple Myeloma, normal kappas and normal lambdas (Data from Figure 3). (0.47 MB TIF) [file pone.0005169.s011.tif]
